# Atypical Presentation of Boerhaave Syndrome With Hypoxia and Unresponsiveness

**DOI:** 10.7759/cureus.27848

**Published:** 2022-08-10

**Authors:** Jordan Bury, Adam Fratczak, Jeffrey A Nielson

**Affiliations:** 1 General Medicine, Kettering Health, Dayton, USA; 2 Emergency Medicine, Kettering Health, Dayton, USA

**Keywords:** esophageal rupture, boerhaave syndrome, thoracostomy tube, esophageal stent, atypical chest pain

## Abstract

The following case discusses the atypical presentation of a spontaneous esophageal rupture that presented as acute hypoxic respiratory failure in the emergency department. The patient initially arrived by ambulance with a chief complaint of non-radiating chest pain for approximately one hour. Within minutes after arrival, the patient became hypoxic and bradycardic, requiring supplemental oxygen. A computed tomography (CT) angiogram of the chest showed a pneumothorax, pneumomediastinum, and left lower lobe consolidations concerning for pneumonia. The patient was resuscitated in the emergency department, and a chest tube thoracostomy was performed. Upon admission to the hospital, an esophagogram with contrast showed an esophageal leak at the gastroesophageal junction with the contrast extending into the left pleural space which required surgical intervention. This case highlights the complicated nature and variable presentations of Boerhaave syndrome and the importance of stabilizing the airway, breathing, and circulation in a decompensating patient even when the etiology is not clear at the time of presentation.

## Introduction

Boerhaave syndrome is a condition in which there is a spontaneous rupture of the esophagus secondary to increased intraesophageal pressure as is commonly seen with vomiting or straining. The condition is very rare, with an incidence rate of approximately 3.1 per 1 million per year. The patients at highest risk for this condition are middle-aged males [1]. Approximately 15% of esophageal rupture cases are spontaneous in nature (i.e., Boerhaave); however, a majority are iatrogenic, typically during or after esophagogastroduodenoscopy (EGD) [2]. Boerhaave syndrome is a grave medical condition and one of the most lethal GI tract disorders with mortality rates approaching 40%. Managing esophageal ruptures continues to be challenging, with goals of treatment centered around controlling the infection combined with fasting and enteral tube feedings [3].

## Case presentation

We present a case of a 50-year-old male with an acute spontaneous esophageal rupture presenting to the emergency department. Emergency medical services (EMS) received a call regarding a middle-aged male with approximately one hour of non-radiating chest pain. The patient described his pain to EMS as severe, sharp, located in the center of his sternum, and non-radiating. The patient initially denied other past medical history or medication history. On route to the hospital, the patient received sublingual nitroglycerin due to an initial history concerning for acute coronary syndrome. This subsequently dropped the patient’s systolic blood pressure from approximately 180 to 100, raising the suspicion of a possible right coronary artery (RCA) occlusion. Upon arrival, the patient was calmly sitting on the stretcher. His vitals were as follows: blood pressure, 120/80 mm Hg; heart rate, 97 beats per minute; respiratory rate: 22 breaths per minute, and oxygen saturation (SaO_2_), 98% on room air, and he was afebrile. As the patient was transferring himself over to the hospital bed, he became severely agitated, clutching his chest and screaming in pain. Within seconds, the patient became obtunded with vital signs revealing hypoxia and bradycardia. After initiation of high-flow nasal cannula (HFNC), the patient’s hypoxia began improving and he was subsequently moved to the resuscitation bay. EKG revealed sinus rhythm without ST elevations. Blood work was obtained which revealed a white blood cell count of 11.3 x10^9^/L and lactic acid of 2.9 mmol/L, and initial troponin was negative.

**Figure 1:**
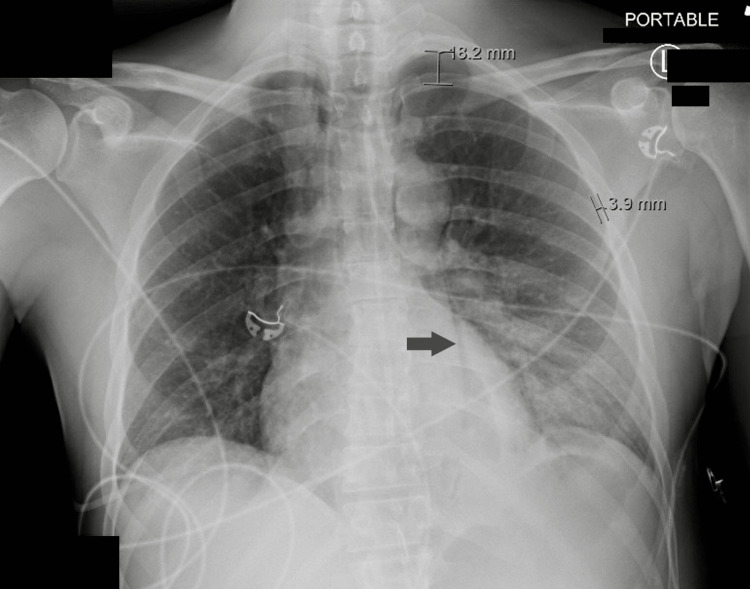
AP portable chest x-ray showing pneumothorax and mediastinal air (arrow) AP: anterior-posterior.

**Figure 2:**
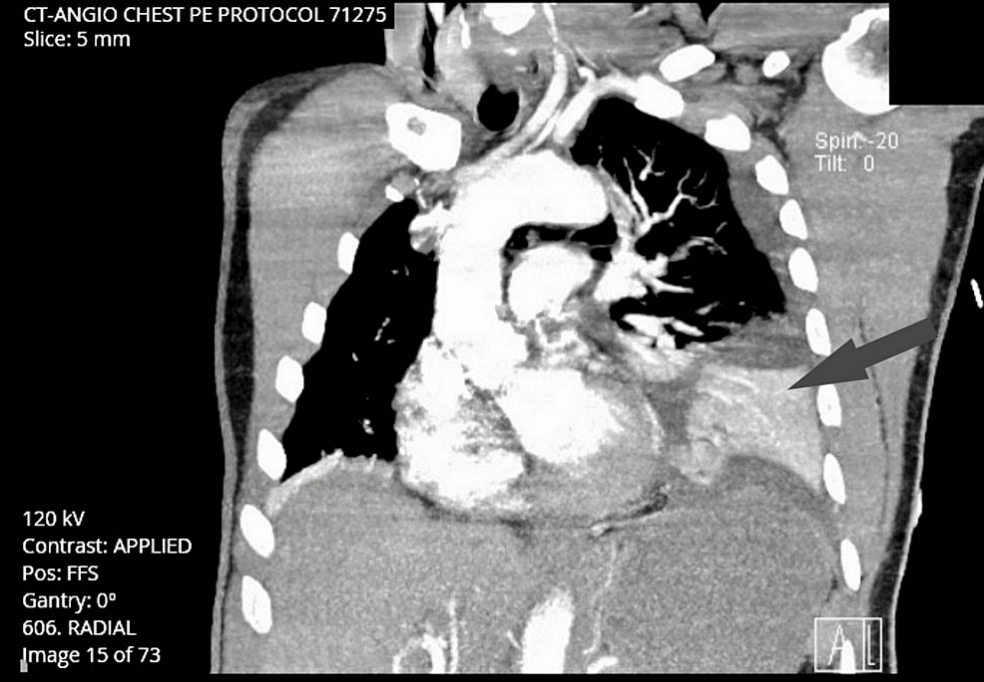
CT angiogram showing pleural effusion (arrow) PE: pulmonary embolism; FFS: feet first-supine.

Initial chest x-ray revealed patchy opacities in the left lower lobe favoring pneumonia. Pneumothorax and mediastinal air were present as well (Figure 1). Given the patient’s acute hypoxic decompensation and episode of unresponsiveness, there was also a concern for a possible pulmonary embolism. The subsequent CT angiogram further characterized the left pneumothorax showing extensive pneumomediastinum and moderate bilateral pleural effusions. The effusion is demonstrated in Figure 2. Chest tube thoracostomy was performed. After placement, approximately 400 mL of serosanguineous fluid along with mucopurulent discharge was evacuated. The purulent discharge collected in the drainage system raised the suspicion of the presence of an empyema. The patient was subsequently started on empiric antibiotics. Shortly after placement of the chest tube, the patient improved and he was able to maintain adequate oxygen saturation on room air and his mentation improved. Serial troponins were negative. The patient was then admitted to the hospitalist team with a consult to surgery for further evaluation of the effusions and mediastinal changes.

**Figure 3:**
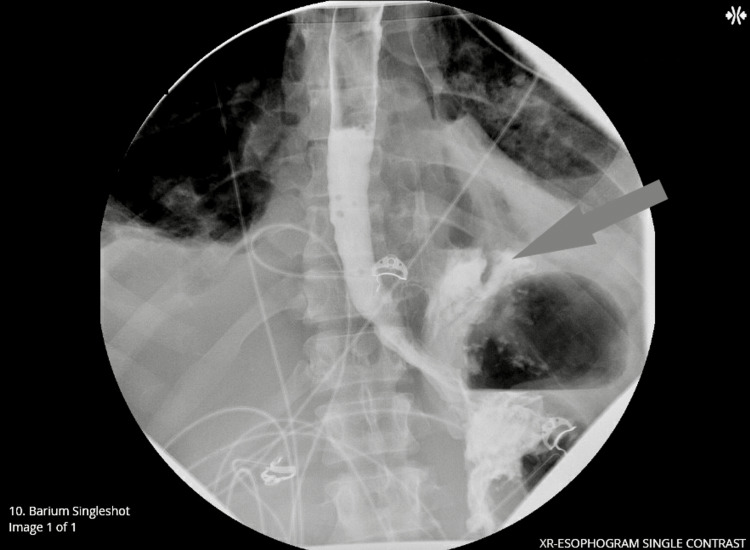
Esophagogram showing contrast leak (arrow) XR: x-ray.

After admission, outside records became available revealing a medical history significant for alcohol abuse, chronic pain, anxiety, hypertension, hyperlipidemia, and asthma. Further imaging with an esophagogram study with oral contrast was ordered to further evaluate the presence of pneumomediastinum which was noted on the initial CT angiogram obtained in the ED. It showed an esophageal leak at the gastroesophageal (GE) junction with contrast extending into the left pleural space cementing the diagnosis of Boerhaave syndrome (Figure 3).

Preoperative antibiotics were dispensed per protocol prior to general anesthesia being administered. Emergent thoracotomy was performed with an incision between the sixth and seventh rib space on the right. A large amount of serosanguineous fluid along with succus was suctioned from the thoracic cavity. Non-viable tissue was debrided. A 4-cm linear perforation was visualized and sutured closed. A myocostal flap was created using the intercostal muscle from the sixth rib. The thorax was then copiously irrigated with vancomycin-impregnated saline, and two 24-French channel drains were placed in the anterior axillary line. Blood loss was estimated at less than 100 cc. The patient remained off oral intake (NPO) after surgery and continued to receive IV antibiotics. Later in the hospital course, due to a persistent esophageal leak, the patient required an EGD for esophageal stent placement. The following day, this required a further revision as the initial stent placement migrated. When the patient was later interviewed, he did admit to a single episode of vomiting one to two hours prior to the chest pain, which he did not mention in his initial presentation in the ED. He remained in the hospital for 35 days, after which he was discharged and is currently doing well tolerating oral feeds.

## Discussion

Boerhaave syndrome is a life-threatening condition that has a high mortality rate even with prompt diagnosis and rapid intervention. A “pooled meta-analysis published in 2013 showed a mortality rate of 12%, while a nation-wide population-based study in England reported a 35% overall mortality rate in patients” with Boerhaave syndrome [4]. Boerhaave is caused by increased intraesophageal pressure leading to a transmural tear through the esophagus. It can occur in a structurally normal esophagus, although the presence of esophagitis and ulcers has been seen in a subset of patients [1]. The transmural tear typically occurs in the left posterolateral aspect of the distal esophagus which can lead to contamination of the mediastinum with gastric contents. This often results in infection with an increased risk for sepsis, leading to organ failure. As highlighted by this case, the complications of an esophageal perforation can be rapid in onset and lead to sudden clinical deterioration.

Diagnosis of Boerhaave syndrome can be challenging; therefore, a thorough history and physical examination should be done. While vomiting is the most common cause of the esophageal rupture, other etiologies include weightlifting, defecation, seizures, trauma, and other events that trigger an increase in intrathoracic pressure must be sought. Individuals with excessive alcohol and excessive food intake are also at an increased risk [4]. These patients may present with vague symptoms or some combination of the classic Mackler triad: vomiting, chest pain, and subcutaneous emphysema. The location and degree of leakage also play a key role in patient presentation.

The preferred diagnostic tests for esophageal perforation include water-soluble contrast imaging with a dynamic swallow study or chest CT with oral contrast. Laboratory tests play a minimal role in the diagnosis, but they do help exclude more common conditions on the differential. Due to its non-specific symptoms, Boerhaave syndrome mimics many cardiopulmonary and gastrointestinal conditions. Chest x-rays have the potential to show findings suggestive of an esophageal rupture (e.g., free air and pulmonary effusions), but the x-ray cannot be used to rule out the diagnosis. Therefore, CT should be used due to its high sensitivity and wide diagnostic ability. In this case, the bilateral pleural effusions present in the imaging were secondary to the extravasation of gastric content into the pleural cavity. They were likely not present earlier when the chest x-ray was performed. The mucopurulent and serosanguineous fluid which was initially misidentified as a possible empyema instead turned out to be the patient’s gastric contents.

Deciding on the best treatment for an esophageal rupture still remains a challenge. There is no best choice, but common treatment modalities include conservative, endoscopic, or surgical. Surgical interventions are divided into esophageal repair through open thoracotomy or video-assisted thoracoscopic surgery (VATS) procedure. If there is significant extraluminal content extravasation or a greater than 24-hour delay in diagnosis, the repair can be additionally reinforced with a vascularized pedicle flap. Typically, these flaps are formed using the intercostal muscles, but the serratus and latissimus dorsi muscles are alternative options as well.

## Conclusions

Even with rapid diagnosis and treatment, Boerhaave syndrome is a potentially lethal gastrointestinal tract disorder. For this reason, it is necessary to have a high index of suspicion. While the preferred diagnostic testing for esophageal perforation includes water-soluble contrast imaging with a dynamic swallow study or CT chest with oral contrast, this is not always feasible. This case highlights the importance of considering esophageal perforation in the differential diagnosis of chest pain regardless of frequency, severity, or even the presence of emesis prior to symptom onset. Additionally, it emphasizes the importance of incorporating a multidisciplinary approach for successful management and recovery for patients suffering from Boerhaave syndrome.
